# Activation of neurogenesis in the hippocampus is a novel therapeutic target for Alzheimer's disease

**DOI:** 10.1002/nep3.25

**Published:** 2023-11-21

**Authors:** Akihiko Taguchi, Yuka Okinaka, Akiko Takeda, Takayuki Okamoto, Johannes Boltze, Carsten Claussen, Sheraz Gul

**Affiliations:** ^1^ Department of Regenerative Medicine Research Foundation for Biomedical Research and Innovation at Kobe Hyogo Japan; ^2^ Department of Pharmacology, Faculty of Medicine Shimane University Izumo Shimane Japan; ^3^ School of Life Sciences University of Warwick Coventry UK; ^4^ Fraunhofer Institute for Translational Medicine and Pharmacology ITMP Hamburg Germany; ^5^ Fraunhofer Cluster of Excellence for Immune‐Mediated Diseases CIMD Hamburg Germany

**Keywords:** Alzheimer's disease, cell‐cell interaction, cell therapy, gap junctions, neurodegenerative disease, neurogenesis

## Abstract

- Targeting single mechanisms of physiological (aging) and pathological (neurodegeneration) loss of function in the brain may not be sufficient.
- Cell–cell interactions between transplanted adult stem cells and resident cells via gap junctions have the potential to support the aging or diseased brain.
- These cell–cell interactions can also increase hippocampal neurogenesis.
- This may be a novel therapeutic strategy for Alzheimer's disease and other neurodegenerative diseases that could be applied alongside any established treatments.

The pathologies associated with age‐related brain dysfunction, including Alzheimer's disease (AD), Parkinson's disease, Lewy body dementia, and vascular dementia, often share similarities across multiple diseases.^1^ While various factors, such as amyloid β, Tau protein, α‐synuclein, chronic inflammation, and impaired cerebral microcirculation have been suggested as potential therapeutic targets, addressing a single factor is unlikely to be sufficient in preventing or treating age‐related brain dysfunction. Drawing from these observations, we postulate that the impairment of brain function associated with aging results from the intricate dysregulation of a variety of biological processes.

A prerequisite for the prevention and development of effective treatments for age‐related brain dysfunction are the elucidation of their underlying biological processes. To facilitate this, mammals with well‐regulated and healthy brain function that align with their wild‐type lifespan (which is known to vary considerably) can be used as model organisms. Thus, correlating biological pathways across different species would be expected to shed light on their ranking and relative contributions toward age‐related impairment of brain function.^2^ We propose that the cause of age‐related brain dysfunction is not merely an accumulation of a multitude of disorders, but rather the natural deterioration of brain functionality once the wild‐type lifespan is traversed when there would normally be no need for the brain to remain in an optimal functional condition (Figure 1A). This hypothesis provides the perspective that general age‐related phenomena, such as accumulation of mitochondrial damage and DNA injury, would also not be the causes but rather the results of aging after wild‐type lifespan when there would be no need to remain in an optimal condition.

**Figure 1:**
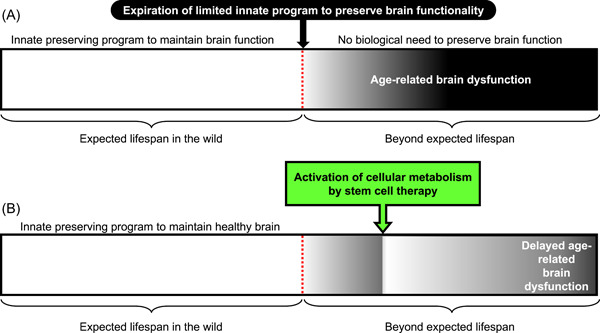
Novel concept to mitigate the age‐related impairment of brain function. (A) Schematic representation of a novel concept underlying the brain dysfunction during aging, caused by expiration of the limited innate program to preserve brain functionality. (B) Novel therapeutic approach to support cellular metabolism by transplanted stem cells via gap junction in the aged brain, mitigating age‐related brain dysfunction.

Despite the complex underlying causes of brain functionality and the essence of senescence being poorly understood,^2^ the idea that the increase in brain dysfunction with aging due to the expiration of the limited innate program to preserve the brain in a healthy condition provides a novel approach for neuroprotection and reversal of brain dysfunction. To this end, we focused on neurogenesis in the adult brain. In the mammalian brain, neuronal stem cells are located in the subventricular zone of the lateral ventricles and subgranular zone of the hippocampus, and the neurogenesis is reduced with aging and pathological disorders, such as AD.^3^, ^4^ Cell‐based therapies, using hematopoietic or mesenchymal stem cells, focusing on enhancement of endogenous neurogenesis have been proposed for the injured brain.^5^ Our research has demonstrated that stem cell transplantation enhances neurogenesis in the hippocampus of aged mice, leading to the restoration of short‐term memory.^6^, ^7^ Moreover, we have identified the fundamental therapeutic mechanism of action of such stem cell therapies that normalize the cellular metabolism of cells with diminished function through gap junction‐mediated direct cell–cell interaction,^8^, ^9^ as gap junction‐mediated cell–cell interactions are notably impaired in the aging brain.^7^ These findings suggest that activating cellular metabolism via gap junction is a novel therapeutic target, given that the metabolism of the aging brain is known to decline.^10^ To this end, stem cell therapy has the potential to prolong the limited innate program to preserve brain functionality, at least partially (Figure 1B). We anticipate that advancements in stem cell biology may result in a significant breakthrough in neuroprotection and new therapies that can reverse age‐related brain dysfunction.

Our recent discoveries have demonstrated that stem cell treatment can enhance neurogenesis in the hippocampus resulting in the restoration of short‐term memory.^6^, ^7^ This provides a firm foundation for novel therapeutic approaches for AD, whose key symptom is short‐term memory impairment by activating neurogenesis in the hippocampus using stem cell therapy. The validity of this approach is corroborated by the independent reports that individuals with AD exhibit considerably less neurogenesis in the hippocampus,^3^ and that neurogenesis in this region of the brain is vital for short‐term memory^11^ and restoration of neurogenesis in the hippocampus may, alongside other interventions targeting the AD pathophysiology, alleviate short‐term memory deficits in AD (Figure 2). We have shown that intravenous hematopoietic stem cell transplantation improves neuronal recovery with enhanced neurogenesis in the murine stroke model.^12^ This was further validated in a clinical trial that showed a significant acceleration of neuronal recovery by stem cell transplantation.^13^ Our positive finding relating to bone marrow‐derived hematopoietic stem cell transplantation in aged mice^6^, ^7^ provide a rationale for initiating a clinical trial of autologous bone marrow‐derived hematopoietic stem cell transplantation in AD patients.

**Figure 2:**
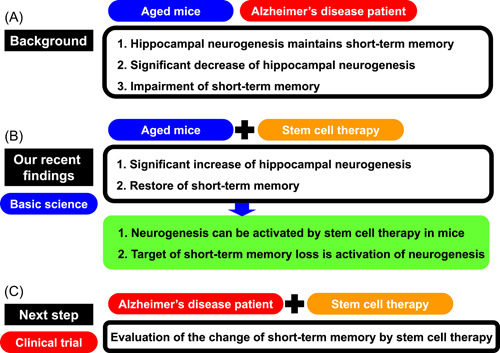
Activation of neurogenesis in the hippocampus as the rational therapeutic target for Alzheimer's disease (AD). (A) Hippocampal neurogenesis is responsible for maintaining short‐term memory, and a significant decrease of hippocampal neurogenesis results in impaired short‐term memory in aged mice and patients with AD. (B) Intravenous hematopoietic stem cell transplantation increased hippocampal neurogenesis and improved short‐term memory in aged mice. (C) Next step is a clinical trial of stem cell therapy to AD patients to evaluate the change of short‐term memory.

It should be noted that further studies will be required to eliminate the inhibitory factor for neurogenesis in the hippocampus, especially, the inhibitory effect of Amyloid β on neurogenesis.^14^ Volumetric magnetic resonance imaging (MRI) analysis of AD patients who received anti‐Amyloid β antibody, Lecanemab, showed a mild effect on suppression of hippocampus volume loss, although the volume loss of the whole brain was significantly accelerated by Lecanemab^15^, ^16^ (Figure 3). These findings support the hypothesis that the rational target for AD is the activation of neurogenesis specifically in the hippocampus, and not neuronal cell death of the entire brain, and a combination of neurogenesis and anti‐amyloid antibody would be more beneficial in AD patients with accumulation of Amyloid β in the hippocampus.

**Figure 3:**
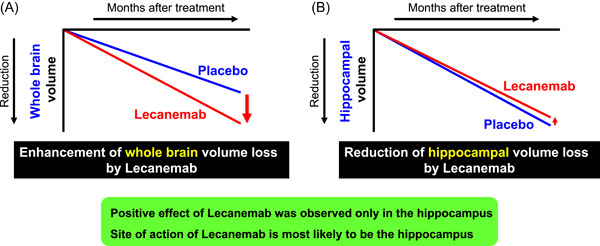
Site of action of Lecanemab is most likely to be hippocampus. Shema of brain volumetric magnetic resonance imaging (MRI) analysis of Lecanemab trial. The analysis showed that Lecanemab significantly enhances whole brain atrophy (A), beside hippocampus (B).

To sum up, it is proposed that age‐related brain dysfunction may not necessarily result from the accumulation of uncontrollable disorders, but rather the natural deterioration of brain function following expiration of the limited innate program to preserve the brain in a healthy condition. We now have identified a means by which this process could potentially be mitigated or even partially reversed by applying stem cell therapy. Furthermore, we propose that the activation of neurogenesis in the hippocampus, also through stem cell therapy, is a promising therapeutic target in AD.

## AUTHOR CONTRIBUTIONS

Akihiko Taguchi, Johannes Boltze, and Sheraz Gul contributed towards writing the manuscript. Yuka Okinaka and Akiko Takeda contributed creation of the concept and figures. Takayuki Okamoto and Carsten Claussen participated in the creation of the concept.

## CONFLICT OF INTEREST STATEMENT

Johannes Boltze is the Editor‐in‐Chief of *Neuroprotection*. He is therefore excluded from the peer‐review process and all editorial decisions related to the publication of this manuscript. The remaining authors declare no conflict of interest.

## ETHICS STATEMENT

The authors have nothing to report.

## Data Availability

Not applicable.
